# Case of Tumor on the Nose, by Mr. Davies

**Published:** 1813-05

**Authors:** D. H. Davies

**Affiliations:** 27, Carburton-street, Fitzroy-square


*To the Editors of the Medical and Physical Journal.*

GENTLEMEN,

OBSERVING a communication in your last Number of Mr. Barlow's, relative to an operation for the removal of a morbid tumor on the nose, allow me to beg insertion of the the following case, as illustrative of the nature of the same disease; and to prove that this morbid excrescence of parts is not altogether an *unexampled* production of nature, nor the idea of removing this troublesome appendage altogether *novel.*

An attempt to prove the causes which produce the many extravagancies which so frequently characterise the works of nature, would at least be hazarding a very intricate and useless inquiry: the laws even that govern natural operations are so very obscure, that the consequences attendant on those operations, or the irregularities that occasionally occur, must be dark indeed.

J. Wingrove, of Luton, Bedfordshire, by trade a cooper, is the subject of the case I intend to state. When I first knew him, he had for several years been inconvenienced by a very *considerable tumor* on the nose, *larger* to appearance than that described by Mr. Barlow's Plate. It was to every appearance what may be strictly termed a *fungous excrescence.* He informed me he had labored under some species of excrescence for above fifteen years, but that for the last seven or ten years it had been increasing considerably in size.

I frequently hinted to him the possibility of removing the tumor; but, like many men, having been accustomed to the evil and inconvenience attending it, he had in some measure become resigned to its presence, and declined any idea of its extirpation.

At length, vanquished by solicitation, aware myself of the benefit he would receive from its extirpation, and the inconvenience he discovered in the prosecution of his trade, so much so that he was totally prevented from ascertaining whether a cask was foul or not, being totally prevented at last from putting the nostrils to the mouth of the cask, he at last consented to the operation, which was performed by myself and another surgeon, who is now deceased.

The patient being seated in a large elbow chair, an incision was made through the centre of the tumor; two lateral incisions were then effected, which, with the assistance of a slight division on the under part, completely removed the major part of the diseased tumor. No arteries of any magnitude made their appearance. The surface of the wound was allowed to bleed freely, and no ligature was found necessary to be applied. It is here perhaps necessary to mention, that two sprouts (if allowed to use the expression) had made their appearance, within the last year, on each side of the original tumor, and it was thought expedient that these should be removed: the scissars were therefore applied to them, and they were entirely extirpated. It may be necessary to mention, that this tumor was not dissected closely to the bone, but that sufficient cutaneous substance was left to admit of the parts ulcerating and cicatrising into a natural form. The tumor when taken off weighed two ounces and a half. I have only to add, the wound perfectly healed in about a month, and no traces whatever were left of the operation; and that when I left the town of Luton, he remained entirely free from any fungous appearance of the nose, and was truly grateful for the benefits resulting from the operation. I must conclude by making one observation, that the likelihood of supervening hæmorrhage ought not to intimidate from performing an operation of this kind, as it is not likely to occur. D. H. DAVIES.

*27, Carburton-street, Fitzroy-square.*

